# Field trial evaluation of the accumulation of omega-3 long chain polyunsaturated fatty acids in transgenic *Camelina sativa*: Making fish oil substitutes in plants

**DOI:** 10.1016/j.meteno.2015.04.002

**Published:** 2015-07-09

**Authors:** Sarah Usher, Richard P. Haslam, Noemi Ruiz-Lopez, Olga Sayanova, Johnathan A. Napier

**Affiliations:** Department of Biological Chemistry and Crop Protection, Rothamsted Research, Harpenden, Herts AL5 2JQ, UK

**Keywords:** Plant metabolic engineering, GM field trials, Omega-3 long chain polyunsaturated fatty acids, Fish oils, Camelina, Oilseeds

## Abstract

The global consumption of fish oils currently exceeds one million tonnes, with the natural *de novo* source of these important fatty acids forming the base of marine foodwebs. Here we describe the first field-based evaluation of a terrestrial source of these essential nutrients, synthesised in the seeds of transgenic *Camelina sativa* plants via the heterologous reconstitution of the omega-3 long chain polyunsaturated fatty acid biosynthetic pathway. Our data demonstrate the robust nature of this novel trait, and the feasibility of making fish oils in genetically modified crops. Moreover, to our knowledge, this is the most complex example of plant genetic engineering to undergo environmental release and field evaluation.

## Highlights

• First demonstration of field stability of EPA and DHA trait in transgenic plants.
• Most complex engineered plant trait to undergo field trialling to date.
• Demonstration of the feasibility of using agriculture to make fish oils.

## 1 Introduction

The consumption of oily fish and the n-3 (also known as omega-3) long chain polyunsaturated fatty acids (LC-PUFA) they contain is now widely recognised by health professionals as conferring an important health benefit, reducing the risk of cardiovascular disease and related metabolic conditions (Saravanan et al., 2010, Swanson et al., 2012). However, the primary source of these nutrients (global oceanic fish stocks) are either at the limits of sustainable management and/or suffering from environmental pollution, precluding increased exploitation (Hixson, 2014). The combination of an expanding human population and changes in dietary consumption (driven by increased affluence) means that more people are consuming animal protein, a significant percentage of which is produced by fish farming (aquaculture) (Cressey, 2009) The aquaculture industry has expanded year-on-year for the last two decades, with 2013 marking the first year in which the majority (51%) of all fish consumed by humans was produced by fish-farming (as opposed to wild capture) (Cressey, 2009, Tacon and Metian, 2009). Thus, although aquaculture has a vital and growing role in the efficient production of food for human consumption, there remains a quandary in the cultivation of marine and salmonid species, namely the requirement for n-3 LC-PUFAs (since such fish, like other vertebrates, cannot effectively synthesise these fatty acids from shorter precursors). Thus, the paradoxical situation exists in which farmed fish must have a dietary source of n-3 LC-PUFAs, primarily derived from other oceanic sources. Supplementation of aquafeed diets with vegetable oils, which lack n-3 LC-PUFAs, results in finished fish devoid of these health-beneficial fatty acids and consumer confusion (Bell et al., 2010).

For all of these reasons, a new sustainable *de novo* source of n-3 LC-PUFAs is desirable, to break the cycle of capture-extraction-feed. We and others have produced genetically engineered plants in which endogenous fatty acid biosynthesis has been augmented with the capacity to synthesise the otherwise non-native n-3 LC-PUFAs (Qi et al., 2004, Wu et al., 2005, Petrie et al., 2012, Ruiz-Lopez et al., 2014). This metabolic engineering demonstrated the feasibility of making eicosapentaenoic acid (20:5n-3; EPA) and docosahexaenoic acid (22:6n-3; DHA) in the seed oils of transgenic plants, and in particular, *Camelina sativa. C. sativa* seed oil has a favourable endogenous fatty acid composition, being rich in the C18 precursor α-linolenic acid (18:3n-3; ALA), and more pertinently, has been shown to be a well-accepted component of aquafeed diets for a range of important fish species (Morais et al., 2012, Hixson et al., 2014). Previously, we and others had shown that *C. sativa* can accumulate up to 20% of seed oil as non-native EPA and DHA, by introducing a suite of algal genes under seed-specific promoters (Ruiz-Lopez et al., 2014, Petrie et al., 2014). These transgenic plants showed no phenotypic difference to their wild type (WT) controls when grown under glasshouse conditions (Ruiz-Lopez et al., 2014) and oil from such plants was an effective replacement for fish oils in salmon feeding trials (Betancor et al., 2015) but for such a potentially important trait to be validated, it is necessary to determine the stability and impact of the altered seed lipid metabolism on plants grown in the field, under real-world conditions. With this in mind, we carried out a field trial on the performance of a genetically modified (GM) *C. sativa* line engineered to accumulate EPA and DHA.

## 2 Materials and methods

### 2.1 Plant material and growth conditions

*C. sativa* grown in the glasshouse (GM-GH) was sown on the 1st of May 2014 and grown in controlled conditions at 25 °C day/16 °C night, 50–60% humidity, and kept under a 16 h photoperiod (long day), with supplemental light provided when ambient levels fell below 400 μmol m^−2^ s^−1^. Harvest usually occurred 95 days after sowing (Supplementary Table 3).

### 2.2 Generation of transgenic plants

Transgenic *C. sativa* lines were generated as previously described (Ruiz-Lopez et al., 2014). The designed vectors were transferred into *Agrobacterium tumefaciens* strain AGL1. *C. sativa* inflorescences were immersed in the *Agrobacterium* suspension for 30 s without applying any vacuum. Transgenic seeds expressing the DHA pathway were identified by visual screening for DsRed activity. Seeds harvested from transformed plants were illuminated using a green LED light. Fluorescent seeds were visualised using a red lens filter. In all cases, no phenotypic perturbation was observed as a result of modification of the seed oil composition.

### 2.3 Vector construction

**Fig. 1:**
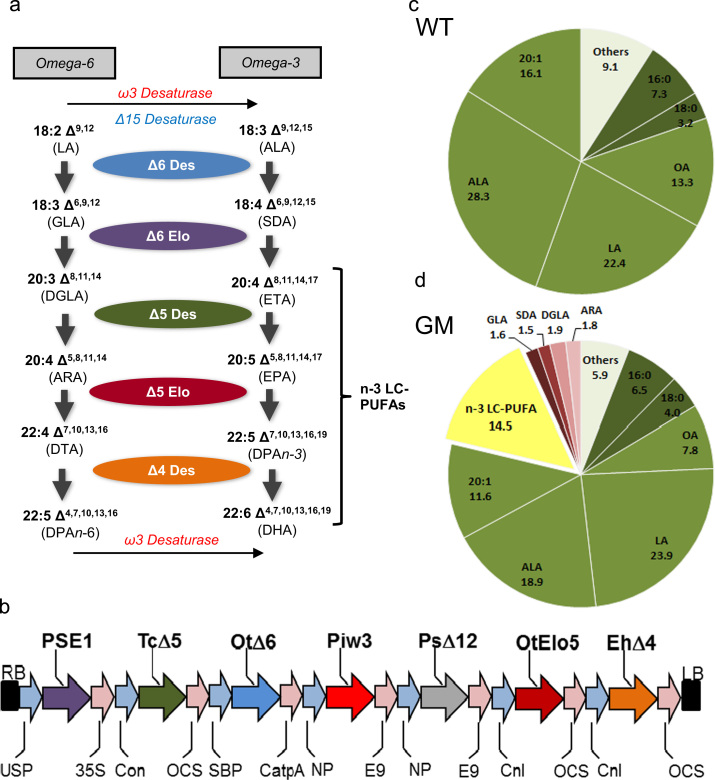
*The biosynthesis of n-3 LC-PUFA in field grown Camelina.* (a) Schematic representation of the enzymatic steps involved in the synthesis of long chain polyunsaturated fatty acids. The individual enzymes are shown, as are the substrates and products. Enzymes recognise both n-3 and n-6 acyl substrates. (b) Schematic representation of the sequences introduced into *C. sativa* to direct the synthesis of EPA and DHA. The individual genes and the activities they encode are described in the Methods section, and are colour-coded according to the pathway in (a). Total fatty acid composition of wildtype (WT) *C. sativa* seeds (c) and the GM line (d) after both were grown in the field. Total n-3 LC-PUFAs are defined as shown in (a), empirically as fatty acids ≥C20 in length, containing four or more double bonds. Abbreviations are defined in (a) or in the text.

The construct used for plant transformation, contained an optimal set of genes for EPA and DHA synthesis (Fig. 1b): a ∆6-desaturase gene from *Ostreococcus tauri*^17^ (Ot∆6) (Domergue et al., 2005a), together with a ∆6 fatty acid elongase gene from *Physcomitrella patens* (PSE1), a ∆5-desaturase gene from *Thraustochytrium* sp. (Tc∆5) (Wu et al., 2005), a Δ12-desaturase gene from *Phytophthora sojae* (Ps∆12) to enhance the levels oflinoleate-CoA (as substrate for the OtΔ6 enzyme) and an ω3-desaturase from *Phytophthora infestans* (Pi-ω3) (Bauer et al., 2012) to increase the conversion of ARA to EPA, OtElo5, an *O. tauri* ∆5 fatty acid elongase gene (Meyer et al., 2004) and Eh∆4, a ∆4-desaturase gene from *Emiliania huxleyi* (Sayanova et al., 2011) both flanked by conlinin promoters and OCS terminators, were added to the p5_EPA construct (Ruiz-Lopez et al., 2013, Ruiz-Lopez et al., 2014). The destination vector contained a DsRed marker for visual selection via seed coat-specific expression of DsRed. All genes were individually cloned under the control of seed-specific promoters, and then combined into a single T-DNA transformation vector as previously described (Ruiz-Lopez et al., 2013). All open reading frames for desaturases and elongases were re-synthesised (GenScript Corporation, NJ; www.genscript.com) and codon-optimized for expression in *C. sativa*.

### 2.4 Field trial

Field experiments were conducted at Rothamsted Research (Harpenden, Hertfordshire, U.K.; grid reference TL 120130; Supplementary Fig. 1). The field trial site consisted of two 5×10 m^2^ subplots of GM *C. sativa* separated by a 2×10 m^2^ strip of WT. The two sub-plots were denoted GM East (GM-E) and GM West (GM-W) (Supplementary Fig. 1). The experimental plot was surrounded by a 7 m-wide WT *C. sativa* strip which served as a “buffer” to mitigate the dispersal of GM pollen. *C. sativa* is considered to be a self-pollinating species, with very low rates of outcrossing or cross-pollination (Walsh et al., 2012). The trial plot was sown on the 15th of May 2014, with T6 GM *C. sativa* seeds sown to create a standing plant density of 290/m^2^, and 300/m^2^ for the WT.

Seedlings were irrigated as necessary following emergence and an insect-proof net was erected around the central experimental plot prior to flowering in order to prevent insect-mediated pollen dispersal; (net removed following the cessation of flowering). Plants were allowed to set seed and monitored for seed maturation. Both WT and GM *C sativa* were harvested on the 5th of September 2014 (113 day growing season), when seeds were fully mature and following three consecutive dry days. In order to complete the harvest, the pollen barrier was removed. To prevent any potential harvest crossover, a 0.25 m-wide strip either side of the border between the GM *C. sativa* and the WT separation was marked and labelled border. Plants within these borders were harvested, but not used for further analysis. The harvested plants were transported to the GM facility glasshouse to further dry before threshing using a Haldrup LT-20 laboratory thresher. Cleaned seeds were tested for DsRed activity, stored, double bagged in paper bags inside locking plastic boxes prior to analysis.

### 2.5 Fatty acid analysis

Total fatty acids in seed batches were extracted and methylated (Garcés and Mancha, 1993). Methyl ester derivatives of total fatty acids extracted were analysed by Gas Chromatography-FID (flame ionisation detection) and the results were confirmed by GC–MS (Ruiz-Lopez et al., 2013). Values presented are from the analysis of single seeds.

### 2.6 Total lipid extraction

Three replicates each from GM-E, GM-W, and WT; consisting of one gram of seeds in 20 mL of chloroform:methanol (2:1) were homogenised using a mortar and pestle. The homogenate was then briefly centrifuged and the liquid phase removed to two separate glass tubes, each made up to a volume of 20 mL chloroform:methanol (2/1). The solvent phase was then washed with the addition of 4 mL of water. After vortexing for 30 s, the mixture was centrifuged at low speed (2000 rpm) to separate the two phases; the lower phase was removed, re-combined and dried under a stream of nitrogen.

### 2.7 Carbon and nitrogen determination

Total carbon and nitrogen were determined by combustion using a Combustion Analyser (LECO TruMac, Leco Corp, St. Paul, MN). This was performed by the in-house analytical unit at Rothamsted Research. Data is present as a percentage of 100% dry matter content.

### 2.8 Thousand grain weight

Three replicates of a thousand seeds from *C. sativa* GM-E, GM-W and WT were counted and the weight determined by mass balance.

### 2.9 Seed water content

Three replicates (1 g of seed) of field grown GM-E, GM-W, WT, GM-glasshouse and WT-glasshouse were dried in an oven (80 °C) until no further weight change could be recorded.

## 3 Results and discussion

### 3.1 Design and approval of the field trial

Appropriate regulatory approval for an experimental environmental release was sought from the UK Ministerial Department for Environment, Food and Rural Affairs (DEFRA), and after independent expert advice, approval was granted in April 2014 (https://www.gov.uk/government/publications/genetically-modified-organisms-rothamsted-research-14r801) with one individual T6 event (DHA-5C♯33_13_4_2) being sown on the dedicated GM trial site on the Rothamsted Experimental Farm. The location and layout of the trial are shown in Supplementary Fig. 1, and the T-DNA construct and its constituent genes and regulatory elements are shown in Fig. 1b, as is the metabolic pathway being reconstituted in transgenic plants (Fig. 1a). Seeds were sown by hand at a defined density of 290/m^2^, and the crop (WT and GM) was allowed to establish without any overt management measures (such as fertilizer or chemical applications). After approximately six weeks, the plants commenced flowering, and by week 16, seed development and maturation was complete. Harvesting was carried out by hand, and after further desiccation in the GM containment glasshouses, seed capsules were mechanically disrupted and threshed. Monitoring of the field material showed no obvious phenotypic differences in the growth, flowering or seed-set of the GM line compared to the WT.

### 3.2 Fatty acid composition of field-grown GM Camelina

The primary aims of this trail were to demonstrate the stability of n-3 LC-PUFA EPA and DHA trait, and also to establish if there was any yield penalty in terms of seed oil or other storage components. The line chosen (based on the availability of seed) did not represent the highest example of a *C. sativa* event accumulating EPA and DHA, but rather an event that represented more moderate levels of these non-native fatty acids. As shown in Supplementary Table 1, the accumulation of these fatty acids was stable for a number of glasshouse (GH)-grown generations, and these data serve as a comparison with the field grown material (with the caveat that the GH conditions are significantly less variable than the field). Total fatty acid methyl esters (FAMes) of seed lipids from field grown line DHA-5C♯33_13_4_2 confirmed the accumulation of n-3 LC-PUFAs to significant levels (14.5%; Fig. 1d), with these fatty acids being completely absent in the field–grown WT *C. sativa* seeds (Fig. 1c). The accumulation of undesirable biosynthetic intermediates (Fig. 1a, d) such as the n-6 fatty acids γ-linolenic acid and arachidonic acid was marginal (<2%) and not increased compared to our previous observations of equivalent GH-grown material (Ruiz-Lopez et al., 2014) confirming the stability of this aspect of the trait when grown in the field.

**Fig. 2:**
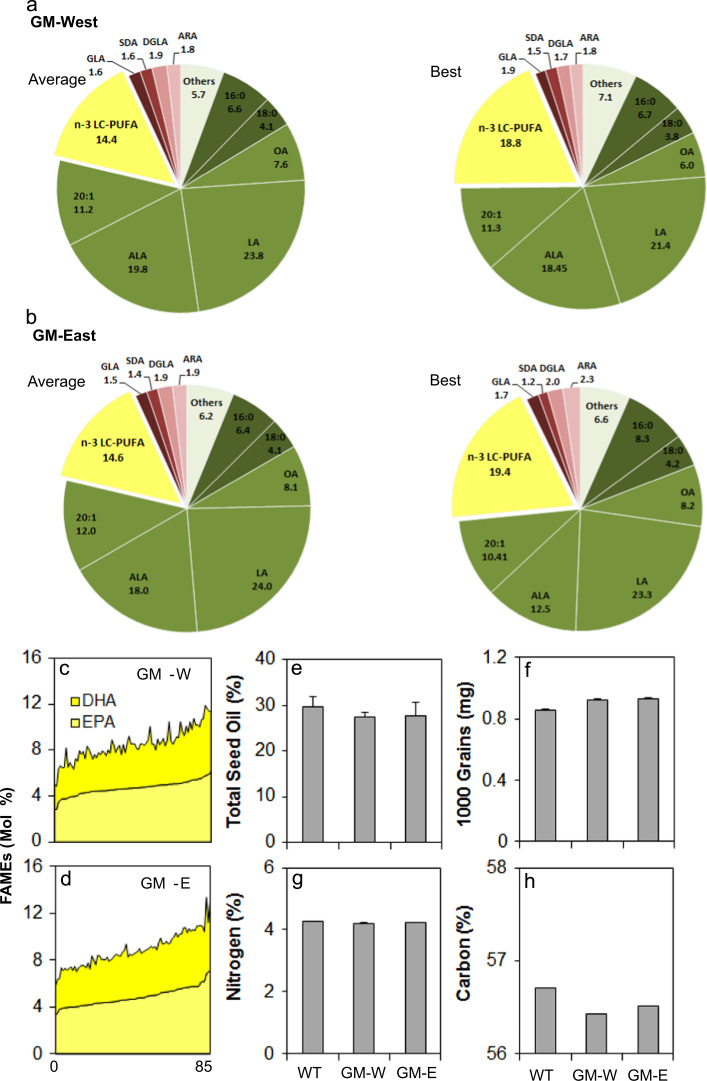
*Seed-specific production of n-3 LC-PUFA in the field.* (a) Average total fatty acid composition of seeds from the GM-West field plot. The highest (best) example is also shown. (b) Average total fatty acid composition of seeds from the GM-East field plot. The highest (best) example is also shown. Total n-3 LC-PUFAs are defined as fatty acids ≥C20 in length, containing four or more double bonds. The levels of specific n-3 LC-PUFAs of interest, EPA and DHA are shown in (c) and (d) for the GM-West and GM-East plots, respectively, based on single seed analysis. See also Supplementary Table for primary data. Total seed oil content is shown in (e), 1000-grain weights (f), total % seed nitrogen (g) and total % seed carbon (h) also shown.

More detailed analysis of the seed total FAMes is presented in Fig. 2, both in terms of variation between the two GM plots (Fig. 2a, b) and specifically in the levels of the two key n-3 LC-PUFAs, EPA and DHA derived from single seed analysis from the two trial plots (Fig. 2c, d; see also Supplementary Table 2). Levels of EPA range from 2.8% to 7.14%, with a similar spread for DHA. For comparison, we carried out the same analysis on GH-grown seeds derived from the same generation as the event as grown in the field. This indicated that there was no significant difference between the levels of EPA and DHA in seeds grown under the two different conditions (Supplementary Fig. 2), confirming the robust nature of the transgenic n-3 LC-PUFA trait. It should be noted that the range and median levels for both EPA and DHA were replicated across the two trial plots, GM-East (E) and GM-West (W), and furthermore the range of EPA/DHA accumulation was overlapping with GM-GH seed levels. The interquartile range (25th to 75th) was marginally lower for both EPA (<2%) and DHA (<1%) in the field grown material compared to GM-GH samples. Given the significant variation in the field conditions, in terms of both temperature and rainfall (detailed in Supplementary Table 3), compared with the optimal, stable conditions of the GH; it is clear that the transgene trait (modified fatty acid composition) is very robust for a range of environmental conditions. In addition, it is well-established that temperature has the potential to influence the overall level of unsaturated fatty acids in seeds (Rochester and Silver, 1983).

### 3.3 Oil content of field-grown GM Camelina

The trial demonstrated that the modified fatty acid composition of field grown material is essentially similar to that of GH-grown lines. In addition, we wished to determine if there was any alteration in oil content arising from the accumulation of these novel fatty acids. We therefore determined the total oil content of seeds from the GM-E and GM-W plots, compared with the WT field plot. As shown in Fig. 2e, there was no significant difference in the total seed oil content between the plots, with all displaying an oil content within the range expected of *C. sativa*. This is (pleasingly) in contrast to other attempts to engineer the accumulation of non-native hydroxylated fatty acids, where a significant decrease in seed oil content is observed via an apparent inhibition of the plastidial fatty acid synthase (Bates et al., 2014). Similarly, total seed nitrogen levels were unchanged in the GM versus WT field material (Fig. 2f), indicating no impact on seed protein content. Interestingly, there was a moderate decrease in total seed carbon (Fig. 2h), whereas the GM seeds yielded a higher 1000 grain weight, compared with the field-grown WT – the reason for this increased mass was predominantly due to higher water content (Supplementary Table 4), though the biochemical basis for this remains to be elucidated.

## 4 Conclusions

Collectively, these data demonstrate for the first time the feasibility of producing fish oils (n-3 LC-PUFAs) in the field. Previous work by us (Ruiz-Lopez et al., 2014) and others (Petrie et al., 2014) have provided clear evidence of the capability to engineer oilseed crops such as *C. sativa* to produce these important fatty acids, but this is the first example of a field-based examination of the stability of the trait and investigation of any yield penalty of oil content. Based on this initial study in which n-3 LC-PUFAs accumulate without any impact on total seed oil, the possibility of making these marine fatty acids via agricultural practices now seems a reality, and fulfilling the hopes and promises made previously (Domergue et al., 2005b, Damude and Kinney, 2007).

## Appendix A Supporting information

Supplementary data **Supplementary Figure 1** Description of the location and layout of the GM *C. sativa* trial. **Supplementary Figure 2** EPA and DHA levels present in GM seeds from plants grown in either field plots or glasshouse. Statistical analysis is as described in Methods. **Supplementary Figure 3** Seed moisture content for field-grown and glasshouse material.

Supplementary data **Supplementary Table 1** Total seed fatty acid composition of selected event DHA-5C♯33_13 over multiple generations.

Supplementary data **Supplementary Table 2** EPA and DHA content (total fatty acid analysis) of single seeds from DHA-5C♯33_13 grown in either the field plots or glasshouse.

Supplementary data **Supplementary Table 3** Recorded weather conditions on the Rothamsted Experimental Farm during the period of the GM field trial. The sowing and harvest dates are also provided, as are the conditions in the glasshouse.

## References

[bib1] Bates P.D., Johnson S.R., Cao X., Li J., Nam J.-W., Jaworski J.G., Ohlrogge J.B., Browse J. (2014). Fatty acid synthesis is inhibited by inefficient utilisation of unusual fatty acids for glycerolipid assembly. Proc. Natl. Acad. Sci..

[bib2] Bauer, J., Qiu, X., Vrinten, P., 2012. Novel Fatty Acid Desaturase and Uses Thereof. US Patent number US20120240248.

[bib3] Bell J.G., Bella J.G., Pratoomyota J., Strachana F., Hendersona R.J., Fontanillasb R., Hebardc A., Guyd D.R., Huntere D., Tocher D.R. (2010). Growth, flesh adiposity and fatty acid composition of Atlantic salmon (Salmo salar) families with contrasting flesh adiposity: effects of replacement of dietary fish oil with vegetable oils. Aquaculture.

[bib100] Betancor M.B., Sprague M., Usher S., Sayanova O., Campbell P.J., Napier J.A., Tocher D.R. (2015). A nutritionally-enhanced oil from transgenic Camelina sativa effectively replaces fish oil as a source of eicosapentaenoic acid for fish. Sci Rep..

[bib4] Cressey D. (2009). Aquaculture: future fish. Nature.

[bib5] Damude H.G., Kinney A.J. (2007). Engineering oilseed plants for a sustainable, land-based source of long chain polyunsaturated fatty acids. Lipids.

[bib6] Domergue F., Abbadi A., Heinz E. (2005). Relief for fish stocks: oceanic fatty acids in transgenic oilseeds. Trends Plant Sci..

[bib7] Domergue F., Abbadi A., Zähringer U., Moreau H., Heinz E. (2005). In vivo characterization of the first acyl-CoA Δ6-desaturase from a member of the plant kingdom, the microalga *Ostreococcus tauri*. Biochem. J..

[bib8] Garcés R., Mancha M. (1993). One-step lipid extraction and fatty acid methyl esters preparation from fresh plant tissues. Anal. Biochem..

[bib9] Hixson S.M. (2014). Fish nutrition and current issues in aquaculture: the balance in providing safe and nutritious seafood, in an environmentally sustainable manner. J. Aquac. Res. Dev..

[bib10] Hixson S.M., Parrish C.C., Anderson D.M. (2014). Use of camelina oil to replace fish oil in diets for farmed salmonids and Atlantic cod. Aquaculture.

[bib11] Meyer A., Kirsch H., Domergue F. (2004). Novel fatty acid elongases and their use for the reconstitution of docosahexaenoic acid biosynthesis. J. Lipid Res..

[bib12] Morais S., Edvardsen R.B., Tocher D.R., Bell J.G. (2012). Transcriptomic analyses of intestinal gene expression of juvenile Atlantic cod (Gadus morhua) fed diets with Camelina oil as replacement for fish oil. Comp. Biochem. Physiol. B.

[bib13] Petrie J.R., Shrestha P., Belide S., Kennedy Y., Lester G., Singh S.P. (2014). Metabolic engineering *Camelina sativa* with fish oil-like levels of DHA. PLoS ONE.

[bib14] Petrie J.R., Shrestha P., Zhou X.R., Mansour M.P., Li Q., Belide S., Nichols P.D., Singh S.P. (2012). Metabolic engineering of seeds with fish oil-like levels of DHA. PLoS ONE.

[bib15] Qi B., Fraser T., Mugford S., Dobson G., Sayanova O., Butler J., Napier J.A., Stobart A.K., Lazarus C.M. (2004). Production of very long chain polyunsaturated ω-3 and ω-6 fatty acids in plants. Nat. Biotechnol..

[bib16] Ruiz-Lopez N., Haslam R.P., Napier J.A., Sayanova O. (2014). Successful high-level accumulation of fish oil omega-3 long chain polyunsaturated fatty acids in an oilseed crop. Plant J..

[bib17] Ruiz-Lopez N., Haslam R.P., Usher S.L., Napier J.A., Sayanova O. (2013). Reconstitution of EPA and DHA biosynthesis in Arabidopsis: iterative metabolic engineering for the synthesis of *n*-3 LC-PUFAs in transgenic plants. Metab. Eng..

[bib18] Rochester C.P., Silver J.G. (1983). Unsaturated fatty acid synthesis in sunflower (Helianthus annuus L.) seeds in response to night temperature. Plant Cell Rep..

[bib19] Saravanan P., Davidson N.C., Schmidt E.B., Calder P.C. (2010). Cardiovascular effects of marine omega-3 fatty acids. Lancet.

[bib20] Sayanova O., Haslam R.P., Venegas-Calerón M., Ruiz-López N., Worthy C.A., Rooks P., Allen M.J., Napier J.A. (2011). Identification and functional characterization of genes encoding the omega-3 polyunsaturated fatty acid biosynthetic pathway from the coccolithophore *Emiliania huxleyi*. Phytochemistry.

[bib21] Swanson D., Block R., Mousa S.A. (2012). Omega-3 fatty acids EPA and DHA: health benefits throughout life. Adv. Nutr..

[bib22] Tacon A.G., Metian M. (2009). Fishing for feed or fishing for food: increasing global competition for small pelagic forage fish. Ambio.

[bib23] Walsh K.D., Puttick D.M., Hills M.J., Yang R.-C., Topinka K.C., Hall L.M. (2012). First report of outcrossing rates in camelina [Camelina sativa (L.) Crantz], a potential platform for bioindustrial oils. Can. J. Plant Sci..

[bib24] Wu G., Truksa M., Datla N., Vrinten P., Bauer J., Zank T., Cirpus P., Heinz E., Qiu X. (2005). Stepwise engineering to produce high yields of very long-chain polyunsaturated fatty acids in plants. Nat. Biotechnol..

