# Supplementary material for: Field trial evaluation of the accumulation of omega-3 long chain polyunsaturated fatty acids in transgenic Camelina sativa: Making fish oil substitutes in plants
Source: Metab Eng Commun. 2015 Jul 9;2:93–8. doi: 10.1016/j.meteno.2015.04.002 (PMC4802427; doi:10.1016/j.meteno.2015.04.002)
Supplement: Supplementary file 1 — Supplementary data Supplementary Figure 1 Description of the location and layout of the GM C. sativa trial. Supplementary Figure 2 EPA and DHA levels present in GM seeds from plants grown in either field plots or glasshouse. Statistical analysis is as described in Methods. Supplementary Figure 3 Seed moisture content for field-grown and glasshouse material. [file mmc1.zip › Supp Figs MEC - Revised.pptx]

## Slide 1
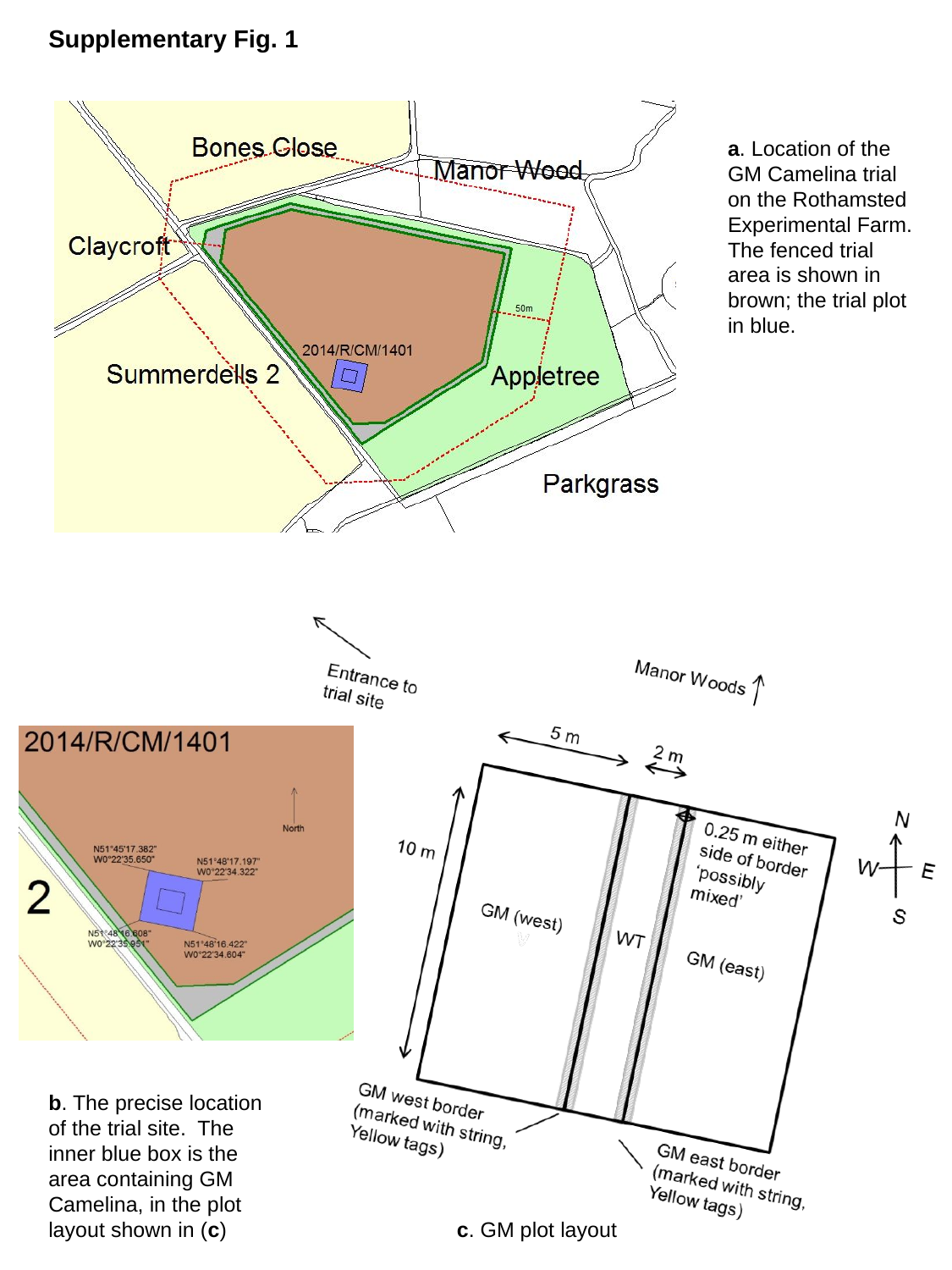

Supplementary Fig. 1
a. Location of the GM Camelina trial on the Rothamsted Experimental Farm. The fenced trial area is shown in brown; the trial plot in blue.
b. The precise location of the trial site. The inner blue box is the area containing GM Camelina, in the plot layout shown in (c)
c. GM plot layout

## Slide 2
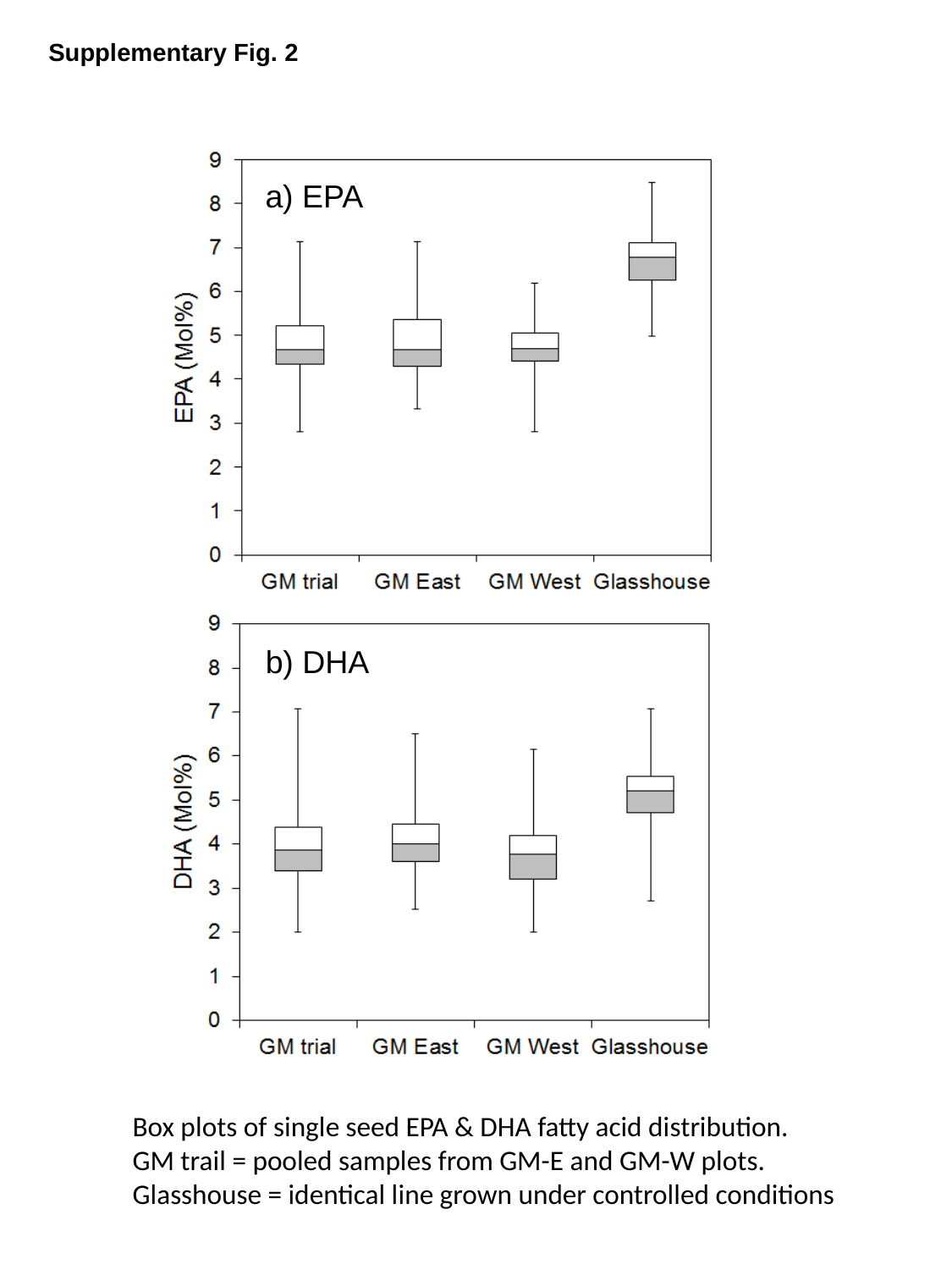

Supplementary Fig. 2
a) EPA
b) DHA
Box plots of single seed EPA & DHA fatty acid distribution.
GM trail = pooled samples from GM-E and GM-W plots.
Glasshouse = identical line grown under controlled conditions

## Slide 3
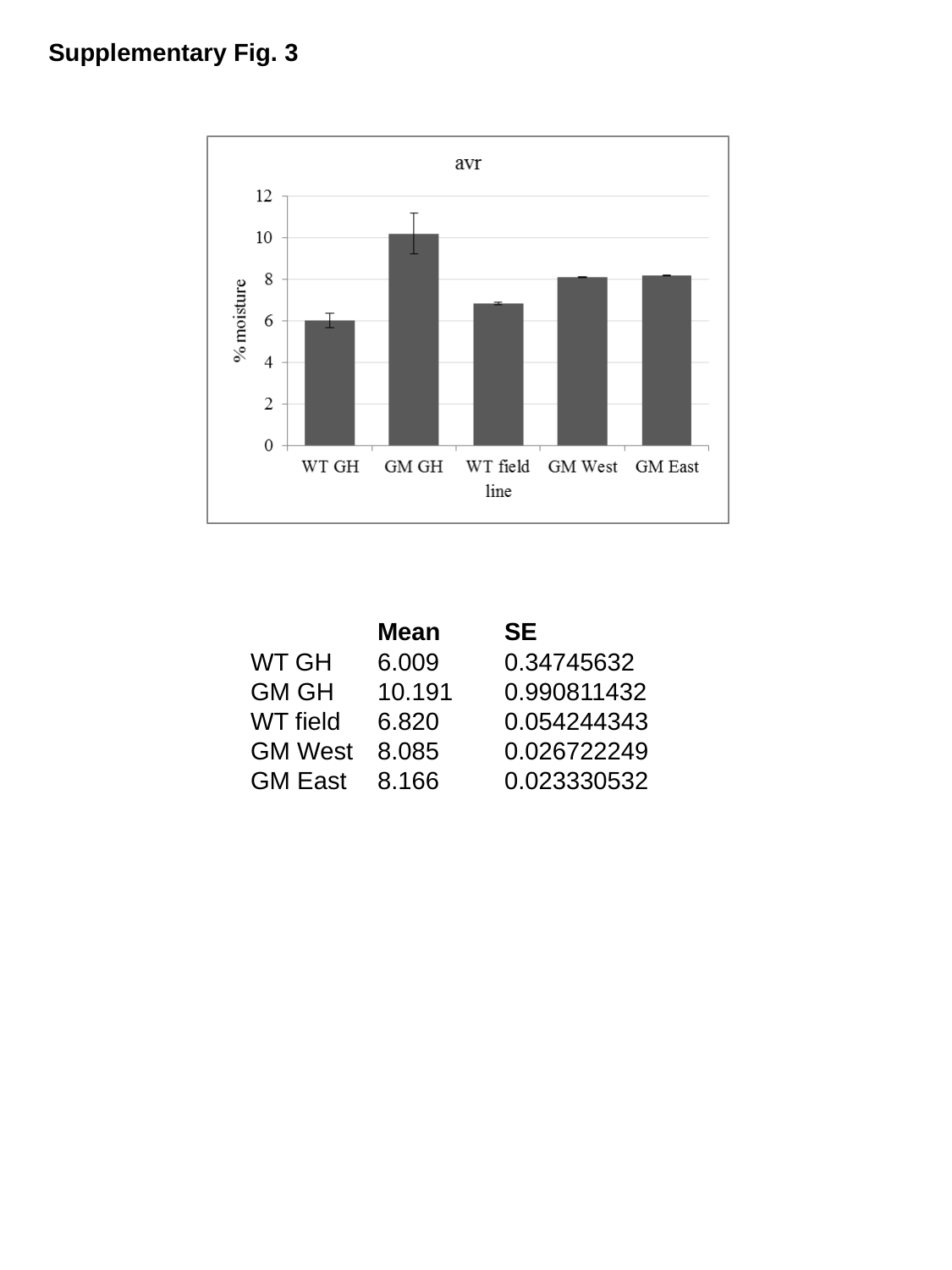

Supplementary Fig. 3
	Mean	SE
WT GH	6.009	0.34745632
GM GH	10.191	0.990811432
WT field	6.820	0.054244343
GM West	8.085	0.026722249
GM East	8.166	0.023330532
