# Supplementary material for: Field trial evaluation of the accumulation of omega-3 long chain polyunsaturated fatty acids in transgenic Camelina sativa: Making fish oil substitutes in plants
Source: Metab Eng Commun. 2015 Jul 9;2:93–8. doi: 10.1016/j.meteno.2015.04.002 (PMC4802427; doi:10.1016/j.meteno.2015.04.002)
Supplement: Supplementary file 2 — Supplementary data Supplementary Table 1 Total seed fatty acid composition of selected event DHA-5C♯33_13 over multiple generations. [file mmc2.pdf]

Fatty Acids

Generation	**Line ID**	16:0	16:1	18:0	18:1	18:2	GLA	ALA	SDA	20:0	20:1	20:2	DHGLA	ARA	20:3n3	20:4n3	EPA	22:0	22:1	DPA	DHA	24:0
	Control_WT	6	0	3	11	19	0	32	0	2	17	3	0	0	2	0	0	0	4	0	0	1
	Control_WT	6	0	3	11	19	0	31	0	2	17	3	0	0	2	0	0	0	4	0	0	1
**T3**	Red-BBC-OtD6-EhD4-33-13	10	9	4	6	23	2	13	1	2	8	1	2	2	1	3	5	0	1	2	6	0
**T4**	Red-BBC-OtD6-EhD4-33-13-x																					
T4	Red-BBC-OtD6-EhD4-33-13-1_a	10	6	4	5	20	3	16	2	1	5	1	1	3	1	3	11	0	1	3	9	0
T4	Red-BBC-OtD6-EhD4-33-13-1_b	11	7	5	4	16	3	13	3	2	5	1	1	2	1	3	13	0	1	4	11	0
T4	Red-BBC-OtD6-EhD4-33-13-1_c	8	3	3	7	21	1	24	1	1	10	1	2	1	1	5	4	0	1	2	5	0
T4	Red-BBC-OtD6-EhD4-33-13-2_a	8	3	5	6	22	1	21	1	2	9	1	2	1	1	5	5	0	1	2	5	1
T4	Red-BBC-OtD6-EhD4-33-13-2_b	8	4	4	6	21	1	21	1	2	9	1	2	1	1	6	5	0	1	2	5	1
T4	Red-BBC-OtD6-EhD4-33-13-2_c	7	3	4	9	25	1	19	1	2	12	2	2	1	1	4	4	0	1	1	3	0
T4	Red-BBC-OtD6-EhD4-33-13-3_a	9	5	4	4	19	1	23	2	2	8	2	2	1	2	6	5	0	1	3	6	1
T4	Red-BBC-OtD6-EhD4-33-13-3_b	9	5	5	5	19	4	14	3	2	6	1	1	2	1	4	12	0	1	3	8	1
T4	Red-BBC-OtD6-EhD4-33-13-4_a	9	5	5	5	21	3	15	3	2	6	1	1	2	0	3	10	0	1	2	6	0
T4	Red-BBC-OtD6-EhD4-33-13-4_b	8	5	4	4	20	1	23	2	2	10	2	2	1	2	5	4	0	2	2	4	0
T4	Red-BBC-OtD6-EhD4-33-13-5_a	9	3	4	3	18	4	17	4	2	6	1	1	2	2	4	14	0	0	3	10	0
T4	Red-BBC-OtD6-EhD4-33-13-5_b	8	4	5	5	19	1	21	2	3	10	1	2	1	1	6	4	0	2	1	4	0
T4	Red-BBC-OtD6-EhD4-33-13-5_c	9	6	5	4	19	3	14	3	3	6	1	0	2	1	4	12	0	0	3	7	0
													Mean				8				6	

T5

	**Line ID**	16:0	16:1	18:0	18:1	18:2	GLA	ALA	SDA	20:0	20:1	20:2	DHGLA	ARA	20:3n3	20:4n3	EPA	22:0	22:1	DPA	DHA	24:0
T5	Red-BBC-OtD6-EhD4-33-13-4-2	8	1	4	8	21	3	14	2	2	8	1	2	2	1	4	8	0	1	2	8	1
T5	Red-BBC-OtD6-EhD4-33-13-4-3	9	0	4	7	20	2	14	2	2	9	1	2	2	1	4	7	0	1	2	8	1
T5	Red-BBC-OtD6-EhD4-33-13-4-4	8	2	5	7	21	3	11	2	2	7	1	1	3	1	3	10	0	1	2	8	0
													Mean				8				8	

T6

	**Line ID**	16:0	16:1A	18:0	18:1	18:2	GLA	ALA	SDA	20:0	20:1	20:2	DHGLA	ARA	20:3n3	20:4n3	EPA	22:0	22:1	DPA	DHA	24:1	
T6	red_BBC_OtD6_EhD4_33_13_5_3	6	0	5	9	24	4	13	2	2	9	1	2	2	1	3	6	0	1	2	5	0	
T6	red_BBC_OtD6_EhD4_33_13_5_3	8	0	5	7	21	4	14	3	2	8	1	1	2	1	3	7	0	1	2	6	0	
T6	red_BBC_OtD6_EhD4_33_13_4_2	7	1	6	7	24	2	12	2	3	9	1	2	2	1	4	6	0	1	2	6	0	Field line
T6	red_BBC_OtD6_EhD4_33_13_4_2	8	1	5	6	22	3	13	2	2	8	1	2	2	1	3	7	0	1	2	7	0	
T6	red_BBC_OtD6_EhD4_33_13_4_2	8	0	5	6	21	3	15	2	2	8	1	2	2	1	3	7	0	1	2	7	0	
T6	red_BBC_OtD6_EhD4_33_13_4_2	8	1	5	5	22	3	13	2	2	8	1	2	2	1	4	7	0	1	3	7	0	
T6	red_BBC_OtD6_EhD4_33_13_4_2	6	0	5	7	24	3	14	2	2	8	1	2	2	1	4	7	0	1	3	7	0	
													Mean				6				6		
T7	red_BBC_OtD6_EhD4_33_13_4_2	8	1	5	7	24	3	12	2	3	8	1	2	3	1	3	7	0	1	2	7	0	GH Con
T7	red_BBC_OtD6_EhD4_33_13_4_2	9	1	4	6	21	3	14	3	2	8	1	1	3	1	3	8	0	1	3	8	0	GH Con
T7	red_BBC_OtD6_EhD4_33_13_4_2	8	1	4	6	22	3	14	2	2	8	1	2	3	1	3	8	0	1	3	8	0	GH Con
T7	red_BBC_OtD6_EhD4_33_13_4_2	8	1	5	6	24	3	11	2	3	8	1	2	2	1	3	7	0	1	3	7	0	GH Con
T7	red_BBC_OtD6_EhD4_33_13_4_2	8	0	5	7	23	3	13	2	2	8	1	2	3	1	3	8	0	1	3	7	0	GH Con
T7	red_BBC_OtD6_EhD4_33_13_4_2	6	0	5	7	25	3	14	2	3	8	1	2	2	1	4	6	0	1	3	6	0	GH Con
T7	red_BBC_OtD6_EhD4_33_13_4_2	8	0	5	6	22	3	15	2	2	8	1	2	3	1	3	7	0	1	2	7	0	GH Con
													Mean				7				7		
