# Supplementary material for: Field trial evaluation of the accumulation of omega-3 long chain polyunsaturated fatty acids in transgenic Camelina sativa: Making fish oil substitutes in plants
Source: Metab Eng Commun. 2015 Jul 9;2:93–8. doi: 10.1016/j.meteno.2015.04.002 (PMC4802427; doi:10.1016/j.meteno.2015.04.002)
Supplement: Supplementary file 3 — Supplementary data Supplementary Table 2 EPA and DHA content (total fatty acid analysis) of single seeds from DHA-5C♯33_13 grown in either the field plots or glasshouse. [file mmc3.pdf]

Camelina Single Seed FAMEs Analysis - EPA & DHA Content (Mol%)

Omega-3 Field Trial_2014 (GM-EW+ GM-E)			GM WEST			GM EAST			GM GlassHouse		
**Seed #**	**EPA**	**DHA**	**Seed #**	**EPA**	**DHA**	**Seed #**	**EPA**	**DHA**	**Seed #**	**EPA**	**DHA**
1	2.8	2.2	1	2.8	2.2	1	3.3	2.5	1	5.0	3.8
2	2.8	2.0	2	2.8	2.0	2	3.5	2.9	2	5.0	3.7
3	3.3	2.5	3	3.5	2.9	3	3.8	2.6	3	5.2	3.8
4	3.5	2.9	4	3.6	3.0	4	3.9	3.5	4	5.2	3.0
5	3.5	2.9	5	3.8	2.7	5	3.9	3.2	5	5.6	3.6
6	3.6	3.0	6	3.8	2.7	6	3.9	3.4	6	5.6	2.7
7	3.8	2.7	7	3.8	4.4	7	4.0	3.2	7	5.6	4.8
8	3.8	2.7	8	3.8	2.7	8	4.0	3.3	8	5.8	4.5
9	3.8	4.4	9	3.9	3.0	9	4.0	3.5	9	5.9	4.6
10	3.8	2.7	10	4.0	2.7	10	4.0	3.1	10	5.9	4.5
11	3.8	2.6	11	4.0	2.4	11	4.0	3.5	11	5.9	4.5
12	3.9	3.5	12	4.0	3.3	12	4.1	3.0	12	6.0	4.4
13	3.9	3.2	13	4.0	3.0	13	4.1	3.3	13	6.0	4.5
14	3.9	3.0	14	4.2	3.8	14	4.1	3.4	14	6.0	4.3
15	3.9	3.4	15	4.2	3.4	15	4.1	3.4	15	6.1	5.2
16	4.0	2.7	16	4.3	3.6	16	4.1	3.4	16	6.1	5.1
17	4.0	3.2	17	4.3	3.0	17	4.1	3.2	17	6.1	4.7
18	4.0	2.4	18	4.3	4.0	18	4.2	3.6	18	6.3	5.4
19	4.0	3.3	19	4.3	3.8	19	4.2	3.1	19	6.3	4.7
20	4.0	3.3	20	4.4	3.3	20	4.2	4.2	20	6.3	4.7
21	4.0	3.5	21	4.4	2.8	21	4.3	3.7	21	6.3	5.9
22	4.0	3.1	22	4.4	3.2	22	4.3	3.3	22	6.5	5.6
23	4.0	3.5	23	4.4	2.9	23	4.3	4.1	23	6.5	5.4
24	4.0	3.0	24	4.4	3.4	24	4.3	4.0	24	6.5	4.7
25	4.1	3.0	25	4.4	3.1	25	4.4	3.7	25	6.5	5.8
26	4.1	3.3	26	4.4	3.5	26	4.4	3.7	26	6.6	4.8
27	4.1	3.4	27	4.5	3.3	27	4.4	3.7	27	6.6	4.9
28	4.1	3.4	28	4.5	3.0	28	4.4	3.9	28	6.6	5.3
29	4.1	3.4	29	4.5	3.5	29	4.4	3.5	29	6.6	4.2

30	4.1	3.2	30	4.5	3.2	30	4.4	3.6	30	6.6	4.9
31	4.2	3.6	31	4.5	4.4	31	4.4	3.7	31	6.7	4.9
32	4.2	3.1	32	4.5	3.9	32	4.5	4.0	32	6.7	5.2
33	4.2	4.2	33	4.6	3.2	33	4.5	4.0	33	6.7	5.2
34	4.2	3.8	34	4.6	3.6	34	4.5	3.9	34	6.7	4.8
35	4.2	3.4	35	4.6	3.8	35	4.6	3.8	35	6.7	6.2
36	4.3	3.6	36	4.6	3.0	36	4.6	4.1	36	6.8	5.0
37	4.3	3.0	37	4.6	3.9	37	4.6	4.2	37	6.8	4.0
38	4.3	3.7	38	4.6	3.2	38	4.6	4.8	38	6.8	5.5
39	4.3	4.0	39	4.6	3.9	39	4.6	3.6	39	6.8	4.2
40	4.3	3.8	40	4.7	4.5	40	4.7	3.9	40	6.8	5.2
41	4.3	3.3	41	4.7	3.9	41	4.7	3.7	41	6.8	5.2
42	4.3	4.1	42	4.7	3.9	42	4.7	3.9	42	6.8	5.9
43	4.3	4.0	43	4.7	3.9	43	4.7	3.9	43	6.8	5.3
44	4.4	3.7	44	4.7	3.5	44	4.8	4.0	44	6.9	6.0
45	4.4	3.7	45	4.7	3.4	45	4.8	4.2	45	6.9	5.5
46	4.4	3.7	46	4.8	3.8	46	4.8	3.8	46	6.9	5.4
47	4.4	3.9	47	4.8	3.8	47	4.8	3.9	47	6.9	5.5
48	4.4	3.3	48	4.8	3.6	48	4.8	4.0	48	6.9	5.9
49	4.4	2.8	49	4.8	3.8	49	4.9	4.0	49	6.9	5.2
50	4.4	3.5	50	4.8	4.1	50	4.9	4.1	50	7.0	5.0
51	4.4	3.2	51	4.8	5.2	51	5.0	4.2	51	7.0	5.5
52	4.4	3.6	52	4.9	3.7	52	5.0	4.0	52	7.1	5.2
53	4.4	2.9	53	4.9	3.7	53	5.0	4.2	53	7.1	6.5
54	4.4	3.4	54	4.9	3.1	54	5.0	3.8	54	7.1	5.0
55	4.4	3.7	55	4.9	4.0	55	5.1	4.4	55	7.1	5.3
56	4.4	3.1	56	5.0	3.5	56	5.2	4.7	56	7.1	5.5
57	4.4	3.5	57	5.0	3.7	57	5.2	4.2	57	7.2	5.5
58	4.5	3.3	58	5.0	4.1	58	5.3	4.1	58	7.2	5.7
59	4.5	4.0	59	5.0	3.4	59	5.3	4.3	59	7.2	5.4
60	4.5	3.0	60	5.0	3.9	60	5.3	4.1	60	7.2	5.4
61	4.5	3.5	61	5.0	5.5	61	5.4	4.1	61	7.3	5.9
62	4.5	4.0	62	5.0	3.8	62	5.4	4.5	62	7.4	5.0

63	4.5	3.2
64	4.5	4.4
65	4.5	3.9
66	4.5	3.9
67	4.6	3.2
68	4.6	3.8
69	4.6	3.6
70	4.6	4.1
71	4.6	3.8
72	4.6	4.2
73	4.6	3.0
74	4.6	3.9
75	4.6	4.8
76	4.6	3.2
77	4.6	3.9
78	4.6	3.6
79	4.7	3.9
80	4.7	4.5
81	4.7	3.9
82	4.7	3.7
83	4.7	3.9
84	4.7	3.9
85	4.7	3.9
86	4.7	3.9
87	4.7	3.5
88	4.7	3.4
89	4.8	3.8
90	4.8	3.8
91	4.8	4.0
92	4.8	3.6
93	4.8	3.8
94	4.8	4.2
95	4.8	3.8

63	5.1	3.5
64	5.1	3.8
65	5.1	4.1
66	5.1	4.0
67	5.1	5.4
68	5.2	4.5
69	5.2	4.1
70	5.2	4.9
71	5.2	4.3
72	5.3	5.1
73	5.4	4.4
74	5.4	5.4
75	5.4	4.8
76	5.4	4.8
77	5.5	4.5
78	5.5	5.1
79	5.7	5.1
80	5.8	6.2
81	5.8	5.8
82	5.9	5.5
83	6.2	5.2

63	5.4	4.6
64	5.5	4.9
65	5.5	4.5
66	5.6	4.7
67	5.6	4.7
68	5.6	5.1
69	5.7	5.1
70	5.7	4.7
71	5.7	5.0
72	5.8	4.7
73	5.8	4.9
74	5.8	4.8
75	5.8	5.1
76	5.8	5.1
77	6.1	4.8
78	6.2	4.7
79	6.2	4.2
80	6.9	6.5
81	7.0	4.2
82	7.1	6.0

63	7.4	5.9
64	7.4	6.8
65	7.4	5.8
66	7.5	4.7
67	7.5	5.6
68	7.7	5.4
69	7.8	5.4
70	8.1	7.1
71	8.2	7.1
72	8.3	6.5
73	8.5	6.3

96	4.8	3.9
97	4.8	4.1
98	4.8	4.0
99	4.8	5.2
100	4.9	3.7
101	4.9	3.7
102	4.9	3.1
103	4.9	4.0
104	4.9	4.1
105	4.9	4.0
106	5.0	3.5
107	5.0	3.7
108	5.0	4.1
109	5.0	3.4
110	5.0	3.9
111	5.0	4.2
112	5.0	4.0
113	5.0	4.2
114	5.0	3.8
115	5.0	5.5
116	5.0	3.8
117	5.1	3.5
118	5.1	3.8
119	5.1	4.1
120	5.1	4.4
121	5.1	4.0
122	5.1	5.4
123	5.2	4.5
124	5.2	4.1
125	5.2	4.7
126	5.2	4.9
127	5.2	4.2
128	5.2	4.3

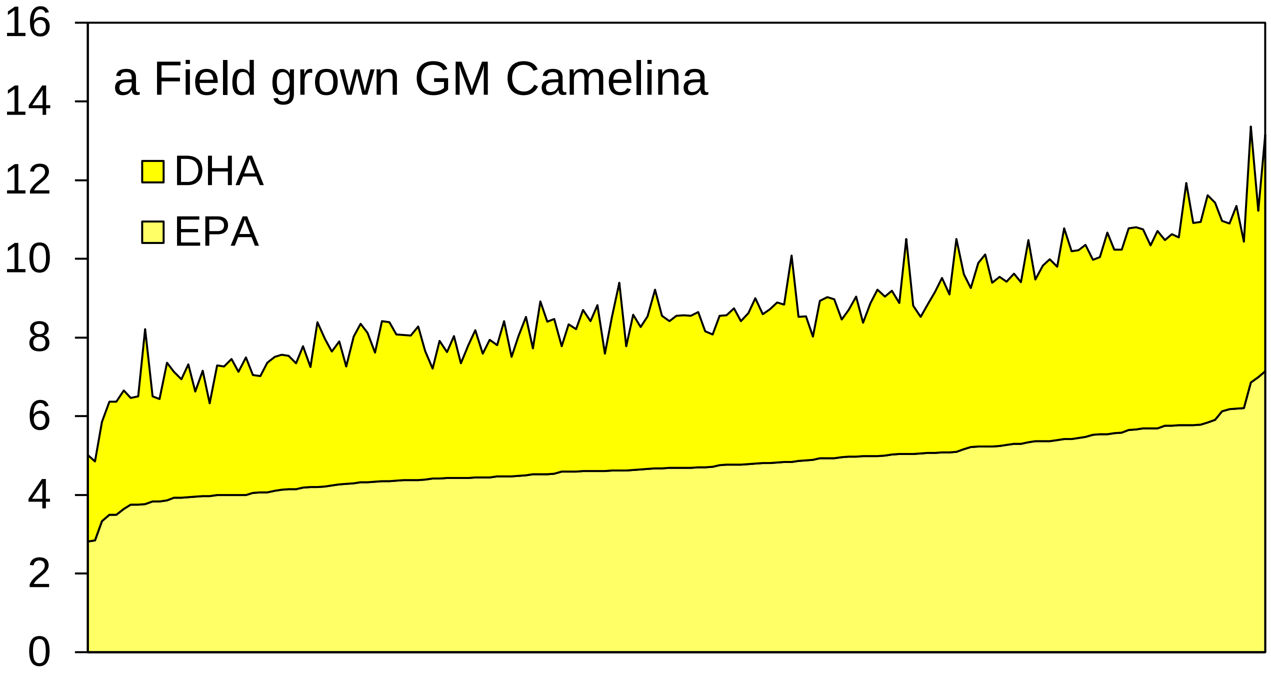

129	5.3	4.1
130	5.3	4.3
131	5.3	4.1
132	5.3	5.1
133	5.4	4.1
134	5.4	4.5
135	5.4	4.6
136	5.4	4.4
137	5.4	5.4
138	5.4	4.8
139	5.4	4.8
140	5.5	4.9
141	5.5	4.5
142	5.5	4.5
143	5.5	5.1
144	5.6	4.7
145	5.6	4.7
146	5.6	5.1
147	5.7	5.1
148	5.7	5.1
149	5.7	4.7
150	5.7	5.0
151	5.8	4.7
152	5.8	4.9
153	5.8	4.8
154	5.8	6.2
155	5.8	5.1
156	5.8	5.1
157	5.8	5.8
158	5.9	5.5
159	6.1	4.8
160	6.2	4.7
161	6.2	5.2

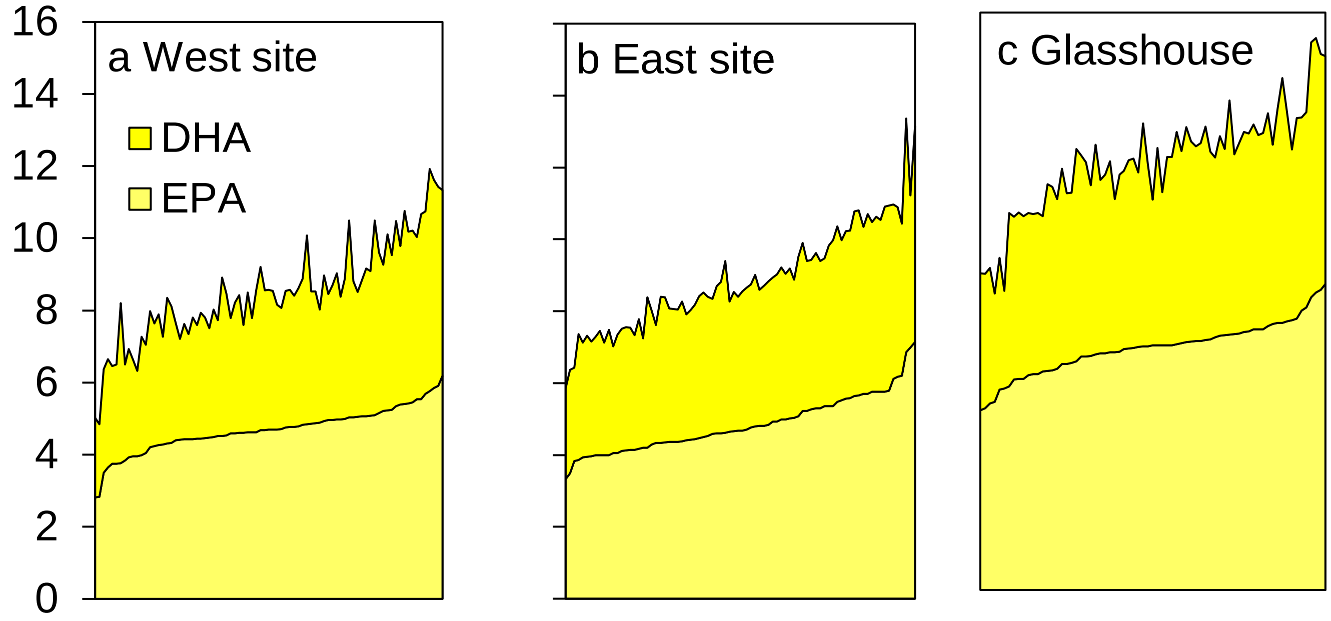

162	6.2	4.2
163	6.9	6.5
164	7.0	4.2
165	7.1	6.0
