# Supplementary material for: Field trial evaluation of the accumulation of omega-3 long chain polyunsaturated fatty acids in transgenic Camelina sativa: Making fish oil substitutes in plants
Source: Metab Eng Commun. 2015 Jul 9;2:93–8. doi: 10.1016/j.meteno.2015.04.002 (PMC4802427; doi:10.1016/j.meteno.2015.04.002)
Supplement: Supplementary file 4 — Supplementary data Supplementary Table 3 Recorded weather conditions on the Rothamsted Experimental Farm during the period of the GM field trial. The sowing and harvest dates are also provided, as are the conditions in the glasshouse. [file mmc4.pdf]

Supplementary Table 3

Rothamsted Experimental Farm
Weather Monthly Summary : 2014 Growing season

	Sunshine		Mean temperatures °C					Frosts	Rain		Rain
			Maximum		Minimum		Dew point		Tipping Bucket		
	Hours	( )	°C	( )	°C	( )	°C		Total mm	( )	days
**May**	173.6	(-20.98)	16.5	(+0.41)	8.3	(+1.39)	9.52	2	82.8	(+28.14)	18
**June**	227.7	(+29.53)	20.1	(+1.01)	10.6	(+0.88)	11.66	0	30.5	(-22.77)	14
**July**	233.4	(+28.25)	23.9	(+2.11)	13.3	(+1.40)	13.69	0	36.9	(-13.00)	14
**August**	168.9	(-27.38)	20.0	(-1.59)	11.4	(-0.44)	11.3	0	113.3	(+49.59)	22
**September**	118.9	(-24.46)	20.1	(+1.85)	11.6	(+1.71)	13.6	0	14.8	(-42.81)	13

Departure from the 30 year means (1981 - 2010) in brackets

Field trial
Sown: 15/05/14, harvested: 05/09/14 on day 113
Flowers started emerging around week 6 –approx day 42

Glass House (GH)

Sown	Harvested	growing days
101: 01/05/14	04/08/14	95
102: 01/05/14	04/08/14	95
103: 01/05/14	05/08/14	96
104: 02/05/14	05/08/14	95
105: 02/05/14	05/08/14	95

The day temp in the glasshouse is 25 degC and night temp is 16 degC.
The compartments also received supplemental lighting whenever the ambient falls below 400 umol-1.
